# Prevalence of Dyslexia among Male Students in Primary Schools and Its Relationship with Obesity and Being Overweight in Ahvaz, Iran

**Published:** 2015-04

**Authors:** Ashrafalsadat Hakim, Alireza Ghorbanibirgani

**Affiliations:** 1 Chronic Disease Care Research Center, Department of Nursing, Ahvaz Jundishapur University of Medical Sciences, Ahvaz, Iran;; 2 Department of Nursing, Gachsaran Branch, Islamic Azad University, Gachsaran, Iran

**Keywords:** Dyslexia, Obesity, Overweight, Student

## Abstract

**Background:**

The most important process in childhood and adolescence is learning. The purpose of this study was to determine the prevalence of dyslexia among primary male school students and the relationship between dyslexia, obesity and overweight.

**Methods:**

This is a cross-sectional study conducted on 1000 male students (first to fifth grade) in primary schools (20 schools) by using the multi-stage random sampling (50 students were selected randomly from each school). Data collection instruments were a weighting scale, a meter for evaluation of obesity and overweight and a reading inventory test for dyslexia. The height and weight were measured based on body mass index (BMI). Data were analyzed using SPSS_17_ by χ² test.

**Results:**

17 and 28 percent of the students were obese and overweight in the first to fifth grades, respectively. On average, the percentage of dyslexia among the unhealthy students was 21 per cent; this rate was 3.5 per cent among the healthy students. In addition, χ² test showed that there was a significant difference between dyslexic and healthy students (P=0.001).

**Conclusion:**

The prevalence of dyslexia among students with overweight and obesity in comparison to healthy students is high; then close monitoring will ensure that these problems are minimized.

## Introduction

About half of the learning disorders are classified in the reading disorders. In most cases, the onset of learning disorders will be determined in pre-school age through second grade. Usually, starting before the first grade indicates a kind of developmental delay in language skills, delay in learning of new concepts at home, or delay in performance in comparison to pre-school and kindergarten peers.^1^ Appearance of the learning disorders early on entry to school is identified in the form of low scores and poor learning. Detection of learning disorders prior to entering to the school is very difficult. Usually, learning problems will recover with treatment, but problems continue on to adulthood with less severity in many children.

Dyslexia is defined as a reading growth disorder. World federation of neurology defines dyslexia as a disorder when a student, despite having a normal intelligence and adequate social and economic facilities, has difficulty reading.^2^ A dyslexic person may have problem in turning written symbols to speech (reading) and expressing words into written symbols (spelling and writing). Furthermore, other linguistic symbols may be damaged such as mathematics.^3^ Because of this type of disorder, dyslexia is better be considered as a problem in processing of information.^4^

As a result of problems in informative processes including poor visual and auditory perception, these children will learn reading harder and later. Sometimes, these children are faced with other problems, such as reversal of numbers, letters and words. Many people with reading disorder show the evidence of lack of progress in the brain cells in regions which can expand the reading disorders in vision and hearing parts. This is related to problems in a brain network that has been formed in the brain and appears to have been specialized for temporal changes.^5^ Incidence of dyslexia in boys is three times more than girls.^6^ According to global statistics, the prevalence of dyslexia is 3.5 to 6 per cent.^4^^,^^7^^-^^8^ In addition, the prevalence of -dyslexia in Iranian 7-10 year old students has been reported 4 to 12 per cent.^9^

As to overweight and obesity, the rates of overweight and obesity among students was reported 12 per cent and 3.2 per cent, respectively.^10^ In another study conducted by Golestan et al., the rate of overweight and obesity was 12.9 and 6.5, respectively.^11^ In addition, in the studies in Columbia state of America,^12^ Kuwait,^13^ Sri Lanka, the South East Asian countries, Mongolia, Kazakhstan, India, Bangladesh, and China, the same results were mentioned.^14^

Obesity and being overweight are also physical problems that involve all people of all ages; especially in childhood, and are threatening the children and adolescents’ health in adulthood period. Incidence of these disorders especially in childhood can cause chronic diseases, like hypertension, cardiovascular diseases, diabetes, kidney diseases and other physical disorders such as learning disorders that can lead to lack of academic achievement in school age children.^15^

There have been studies about dyslexia and learning disorders among children in Iran and other countries. In a study performed with the aim of investigating the prevalence of dyslexia in the normal first grade students in five elementary schools in Isfahan, it was determined that 10 per cent of the five graders had dyslexia, of whom 66 per cent were male and 34 percent female.^16^ In another study, dyslexia was observed in 10.4, 6.8, 5.6 and 4.3 per cent of the second to fifth grade students, respectively.^17^ In another study conducted in 2000, the prevalence of speech disorders in school children in Zanjan was investigated among 1170 students; it was revealed that 29 students (2.4 per cent) had stuttering, 76 students (6.4 per cent) had articulation disorder, 6 students (0.5 per cent) had speech disorder, and one of them (0.08 per cent) had speech delay.^18^ In addition, in a prospective study that was performed in Uromia in 2000 with the aim of investigating the prevalence of dyslexia in the third grade classes, 2067 male and female students from 60 third grade classes were selected; the prevalence of dyslexia among them was estimated 3.2 per cent.^19^

Swanson and colleagues (2003) reported the highest prevalence of dyslexia in the second grade students about 12 per cent and the lowest in the fifth grade students about 3 per cent.^20^ Silver and Hagin in 2002 reported the prevalence of dyslexia in boys to be 8.2 per cent and 4.3 per cent among girls s. Furthermore, the prevalence of dyslexia reported in the fourth and fifth grade students was 3.1 per cent, in the fifth grade boys 8.2 per cent, in the second and third grade male students 10 per cent, in the second and third grade female students 6 per cent, 12 per cent in villages, and 4.8 per cent in cities.^21^ According to a study in Malaysia in 2000, the prevalence of dyslexia was 7 per cent among 200 primary students.^22^

Noshpitz et al. estimated the learning disorder between 4.1 to 14.3 per cent in Germany.^23^ Also, Stanovich et al. reported the prevalence of reading disorder 10.8 percent in rural students, 4.9 percent in urban areas, 9 per cent in boys and 3.2 per cent in female students, and found that the prevalence of reading disorder in male students is three to four times more than female students.^24^ The results regarding the learning disorders vary, but the prevalence of dyslexia is high and attention to this group of disorders has a great importance for providing medical intervention, education and use of rehabilitation services in time.

However, there is new evidence that the nutritional status, especially obesity, is involved in children’s learning disabilities. Support for this theory comes from evidence of poor performance of dyslexics in a variety of motor, time estimation and balance tasks.^25^ Brain imaging studies have also shown anatomical, metabolic and activation differences in the cerebellum of dyslexics.^26^ Finally, this commentary justifies and encourages the necessity of research on the subject. In addition, considering the importance of this issue and lack of studies in this field throughout Iran and Ahvaz, the researchers of this study aimed to determine the prevalence of dyslexia in primary school male students and its relationship with obesity and overweight in 2012-13.

## Materials and Methods

This study is a cross-sectional research on male primary school students (first to fifth grade) from September to November 2012-13 in Ahvaz-Iran. According to the data provided by the Department of Education and Training of Khuzestan province, there were 200 primary schools in the city of Ahvaz, from which 20 were chosen through multistage random sampling method. Approximately, the number of all male students in the primary schools in Ahvaz in 2012 was 50000. This study involved 1000 students and these samples were confirmed through a pilot study at the early stage of the research. After obtaining the necessary approval from the education and training centre and catchment’s area, 1000 students were selected. First, primary schools were listed and then, by means of using random number table, 20 schools in Ahvaz-Iran were selected. Then, 50 students were selected randomly from each school. Inclusion criteria were having normal intelligence (based on teachers’ comments) and having a healthy sense of hearing and vision (according to information contained in students’ records). To ensure the integrity of hearing and vision senses of students, the information on educational and health records were observed and their teachers were consulted for more accuracy. In addition to the students’ record information, students were excluded if teachers expressed suspicious points about them. 

Firstly, for measuring the overweight and obesity, students stood on weighting scale with minimal clothing and without shoes on; the weighting scale was calibrated regularly before and during each sampling. Then, we measured the height. Based on body mass index (BMI), the overweight and obese students were separated from healthy ones.

Secondly, for evaluating dyslexia, a reading inventory test made by Shafiei and colleagues in Isfahan was used.^27^ In order to run the test, individual students stayed in separate rooms, read the related test text loudly. The number of errors was calculated and the students’ comprehension score was calculated by a speech therapist. If a student read less than 90 per cent of words correctly and his/her comprehension score was less than 50 per cent (the errors were more than ten and answers less than two questions) was considered as dyslexic. For finding the correlation between being overweight and obese with dyslexia, the students were divided into two groups (students with and without obesity). Then, the rate of dyslexia was evaluated among each group and the two groups were compared. Written consent was obtained from the parents of students who participated in the study. Finally, the data were entered into pre-designed tables in which the number of errors and comprehension scores was included.

A descriptive analysis was conducted using percentage of quantitative variables and frequencies for qualitative variables, respectively. Differences of quantitative variables between dyslexic and non-dyslexic children were examined through χ² test. All P values were two-tailed with a significant level at 0.05 and all statistical analyses were carried out in SPSS 17.0. 

## Results

The rate of being overweight among students in the fifth grades was 55, 41, 17, 14 and 13 percent, and that of obesity was 23, 34, 11, 9 and 8 percent, respectively. Also, results of reading inventory test among students who were overweight and obese showed that 20, 33, 17, 11 and 7 percent of students in the first to fifth grades were dyslexic, respectively. The number of overweight and obese students   is indicated in Table 1, and Table 2 shows the prevalence rate of dyslexia among overweight and obese students. 17 and 28 per cent of overweight and obese students were in the first to fifth grades, respectively. Furthermore, the rate of dyslexia among the first to fifth grades overweight and obese students was 20 percent in the first grade, 33 percent in the second grade, 17 per- cent in third grade, 11 percent in the fourth grade, and 7 percent in the fifth grade.

**Table 1:** Distribution of the number of overweight and obese students (n=1000)

** Disorder** **Grades **	**Healthy**		**With Overweight**		**Obese**		**Total**	
	**Number**	**Per cent**	**Number**	**Per cent**	**Number**	**Per cent**	**Number**	**Per cent**
First	44	22	110	55	46	23	200	100
Second	50	25	82	41	68	34	200	100
Third	144	72	34	17	22	11	200	100
Fourth	154	77	28	14	18	9	200	100
Fifth	158	79	26	13	16	8	200	100
Total	550	65	280	28	170	17	1000	100

**Table 2:** The prevalence rate of dyslexia among overweight and obese students

** Disorder** **Grades **	**Overweight**		**Obesity**		**Dyslexia**	
	**Number**	**Percent**	**Number**	**Percent**	**Number**	**Percent**
First	110	55	46	23	30	20
Second	82	41	68	34	50	33
Third	34	17	22	11	10	17
Fourth	28	14	18	9	5	11
Fifth	26	13	16	8	3	7
Total	280	28	170	17	98	21
P value	0.001					

On average, the percentage of dyslexia among unhealthy students was 21 percent and this was 3.5 per cent among the healthy students (Table 3). In addition, χ² test shows that there is a significant difference between dyslexic and healthy students (tables 2 and 3) (P=0.001).

**Table 3:** The prevalence rate of dyslexia among students who were not overweight and obese (healthy)

** Disorder** **Grades **	**Healthy**		**Dyslexic**	
	**Number**	**Percent**	**Number**	**Percent**
First	44	22	5	11
Second	50	25	8	16
Third	144	72	4	3
Fourth	154	77	2	1.5
Fifth	158	79	1	1
P value	0.001			

## Discussion

The present study was done to evaluate the prevalence of dyslexia in primary school male students and its relationship with obesity and overweight.  The findings of this study showed that the prevalence of dyslexia among different grades is varied. According to the results, the highest rate of dyslexia was in the first and second grades; this is probably because students in these grades have not found full ability to read and this factor may cause more reading problems. They might need more time to identify and decode words and understand the meanings and concepts. In dyslexic persons, symbols of reading and writing may also be damaged.^28^

According to the results, the rate of dyslexia among unhealthy students was 21 per- cent. One of the causes of the high prevalence of speech disorder in this study was the bilingual community. Prevalence of dyslexia is low in the fourth to fifth grades, indicating the mastery in basic skills in reading in these grades. This is partly justified why the speed of reading and writing in the first and second grades students is low and they need more time to do homework. On the other hand, reading materials in these grades mostly include textbooks that are taught in school, but in the fourth to fifth grades students will both extend the use of the non-academic books and expand their desire to read non-academic books. Swanson et al.^20^ have reported that students in the first, second and third grades, due to lack of vocabulary in comparison to the higher grades, mostly have problem in reading; this will reduce by increased age and receiving further training. Logan and Getchell suggest that children with dyslexia are potentially exposed to being overweight and obese.^29^

On the other hand, research findings showed that 11, 16, 3, 1.5 and 1 percent of the students who are not overweight and obese in the first to fifth grades are dyslexic, respectively, and this findings is close to those of Rahimian Boogar et al.,^17^ reporting 10.8 percent, 9.5 percent, 8.2 percent and 6.9 percent  prevalence of dyslexia in boys in the second to fifth grades, respectively. Furthermore, findings of this study are similar to those of Stanowich et al.^24^ who estimated the prevalence of dyslexia in primary second grades to be 11.4 per cent. In this research, the rate of dyslexia among students who were overweight and obese in the first to fifth grades was estimated 20, 33, 17, 11 and 7 percent, respectively; this shows the high difference in comparison to students who are not overweight and obesity.

According to Table 3, the rate of dyslexia among 550 students not being overweight and obese is 3.5 percent (65 cases), and dyslexia among 450 overweight and obese students was 21 percent (98 cases). Sun et al. reported the prevalence of reading disorder 3.9 per cent and expressed that the levels of BMI in both groups (with and without dyslexia) were not much different.^30^ The study results of Logan and Getchell showed the relationship between motor skills, dyslexia, and body composition, and a significant correlation between BMI and motor skills in children with dyslexia suggests that at least some of these children have problems associated with larger body composition and motor skills.^29^

In this study, there are some limitations in this study such as lack of related evidence disproportionate test location of the schools, and the impossibility of assessment of dyslexia with some of important variables in this disorder like the rate of utilization of media, computer and educational materials at home. It is recommended that these limitations should be considered in further studies. Furthermore, with regard to the epidemiology of dyslexia in Ahvaz, the current results can be generalized more to bilingual regions. We recommended that with doing longitudinal research on girls and boys and their comparison with the healthy persons, we can examine the changes in various aspects. The results of this study can help the nurses as the health care providers in the early diagnosis and appropriate referral of students with dyslexia. 

## Conclusion

The results showed that the prevalence of dyslexia in primary school students, especially among those who are overweight and obese is very high and it requires rapid and timely measures to be taken. Finally, this commentary justifies and encourages further research on the subject. 
